# Mechanism of imidazolium ionic liquids toxicity in *Saccharomyces cerevisiae* and rational engineering of a tolerant, xylose-fermenting strain

**DOI:** 10.1186/s12934-016-0417-7

**Published:** 2016-01-20

**Authors:** Quinn Dickinson, Scott Bottoms, Li Hinchman, Sean McIlwain, Sheena Li, Chad L. Myers, Charles Boone, Joshua J. Coon, Alexander Hebert, Trey K. Sato, Robert Landick, Jeff S. Piotrowski

**Affiliations:** DOE Great Lakes Bioenergy Research Center, University of Wisconsin-Madison, Madison, WI 53726 USA; RIKEN Center for Sustainable Resource Science, Wako, Saitama, Japan; Department of Computer Science and Engineering, University of Minnesota-Twin Cities, Minneapolis, MN USA; Terrence Donnelly Centre for Cellular and Biomolecular Research, University of Toronto, Toronto, ON Canada; Biomolecular Chemistry, University of Wisconsin, Madison, WI USA; Departments of Biochemistry and Bacteriology, University of Wisconsin, Madison, WI USA

**Keywords:** Chemical genomics, Ionic liquids, Lignocellulosic, Biofuel, Biocatalysts

## Abstract

**Background:**

Imidazolium ionic liquids (IILs) underpin promising technologies that generate fermentable sugars from lignocellulose for future biorefineries. However, residual IILs are toxic to fermentative microbes such as *Saccharomyces cerevisiae*, making IIL-tolerance a key property for strain engineering. To enable rational engineering, we used chemical genomic profiling to understand the effects of IILs on *S. cerevisiae.*

**Results:**

We found that IILs likely target mitochondria as their chemical genomic profiles closely resembled that of the mitochondrial membrane disrupting agent valinomycin. Further, several deletions of genes encoding mitochondrial proteins exhibited increased sensitivity to IIL. High-throughput chemical proteomics confirmed effects of IILs on mitochondrial protein levels. IILs induced abnormal mitochondrial morphology, as well as altered polarization of mitochondrial membrane potential similar to valinomycin. Deletion of the putative serine/threonine kinase *PTK2* thought to activate the plasma-membrane proton efflux pump Pma1p conferred a significant IIL-fitness advantage. Conversely, overexpression of *PMA1* conferred sensitivity to IILs, suggesting that hydrogen ion efflux may be coupled to influx of the toxic imidazolium cation. *PTK2* deletion conferred resistance to multiple IILs, including [EMIM]Cl, [BMIM]Cl, and [EMIM]Ac. An engineered, xylose-converting *ptk2*∆ *S. cerevisiae* (Y133-IIL) strain consumed glucose and xylose faster and produced more ethanol in the presence of 1 % [BMIM]Cl than the wild-type *PTK2* strain. We propose a model of IIL toxicity and resistance.

**Conclusions:**

This work demonstrates the utility of chemical genomics-guided biodesign for development of superior microbial biocatalysts for the ever-changing landscape of fermentation inhibitors.

**Electronic supplementary material:**

The online version of this article (doi:10.1186/s12934-016-0417-7) contains supplementary material, which is available to authorized users.

## Background

Biomass-derived fuels and chemicals promise a suite of sustainable bioproducts from future lignocellulosic refineries. Before lignocellulose can be transformed to fuels or chemicals by microbes, however, cellulose and hemicellulose polymers must be converted to fermentable sugars by chemical deconstruction, enzymatic deconstruction, or a combination of both. These deconstruction methods typically generate hydrolysates with toxic small molecules that arise from residual deconstruction chemicals or biomass-derived inhibitors [1, 2] and slow fermentation rates at a substantial economic cost [3].

Lignocellulose- and deconstruction-derived inhibitors come in many forms, and the landscape of these inhibitors is continually changing as new pre-treatment, hydrolysis, and feedstocks technologies are developed [1]. These inhibitors include small acids, phenolics, and furans derived from hemicellulose or lignin and are ubiquitous challenges to bioconversion [2, 4]. However some of the most promising deconstruction methods rely on solvents like ionic liquids (IL) or γ-valerolactone [5, 6], which are partially retained in the hydrolysates and are not readily tolerated by fermentative microorganisms [7, 8].

Despite their toxicity, ILs hold special promise because they can be used either to solubilize crystalline cellulose for enzymatic hydrolysis [9, 10] or to support complete chemical deconstruction without the need for enzymes [6, 11]. Among these ILs, imidazolium ionic liquids (IILs) (e.g. [EMIM]Cl, [BMIM]Cl, [EMIM]Ac) have been the best studied [6, 7, 10]. IILs used for lignocellulose deconstruction are salts composed of organic cation and inorganic anion that are liquids at near ambient temperatures. Adoption of IILs for lignocellulose biorefineries has been slowed by their expense; however, scale-up in production and routes to renewable ILs produced from lignin are likely to surmount this barrier [9, 11]. Maximal recovery and recycling of IILs from lignocellulosic hydrolysates is generally deemed necessary to achieve economic feasibility. Despite a number of recovery efforts, however, IIL-derived hydrolysates can contain ≥1 % residual IIL [8], which is problematic given IIL toxicity to fermentative microbes at 0.01 % [7, 8]. Increasing microbial tolerance of IILs is one strategy to lower the economic cost of IIL-based conversion processes.

The mechanism of IIL toxicity remains unknown in yeast and bacteria. A recent study found that IILs induced a shift from respiration to fermentation in yeast, suggesting they may affect central metabolism or mitochondrial processes [12]. Further, this toxicity could be synergistic in effects with other inhibitors and end-products like ethanol. Defining the mechanisms of IIL toxicity in microbes and identifying gene targets are prerequisites for engineering IIL-tolerant microbes for use in future lignocellulosic biorefineries.

There are several potential routes to create tolerant microbes, including screening natural genetic diversity in strains [13] and directed evolution [14], but these approaches do not necessarily provide enabling knowledge about the mechanisms of tolerance that could be used to engineer the microbes used in specific applications. Chemical genomics offers a third route to tolerant micobes, which is rapid and can be informative about mechanism [15]. This reverse-genetics technique leverages genome-wide mutant collections that can be challenged with a compound, and the fitness of individual mutants assess in a massively parallel way using mutant-specific molecular barcodes [16, 17]. Identification of sets of mutants with specific sensitivities can then give insight into the mechanisms of toxicity. Conversely, resistant mutants can identify points of engineering for tolerance in other genetic backgrounds.

**Fig. 1:**
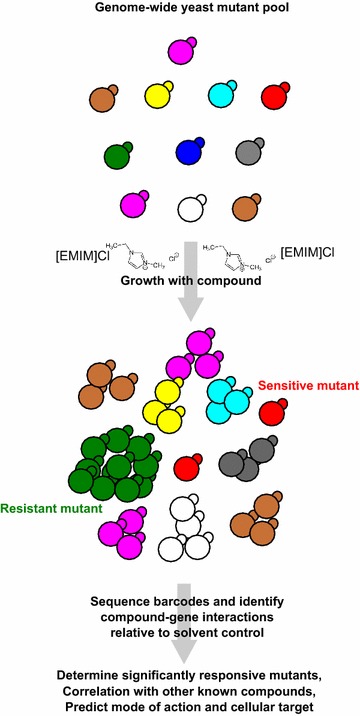
Chemical genomic profiling of ionic liquids. For chemical genomic profiling a genome-wide set of deletion mutants are challenged with a specific compound or solvent control and grown as a pool for several generations. Mutant specific barcodes are then sequenced and compared to control conditions to determine mutants significantly responsive to the chemical stressor (chemical genetic interaction score), which are then used to predict mode of action and points for engineering tolerance

In this report, we describe a chemical genomics approach (Fig. 1) to discover the genome-wide response to IILs toxicity and define their mode of toxicity. Using this information, we identified specific genes that mediate toxicity, identified a mode-of-action of IIL toxicity, and engineered an IIL-tolerant, xylose-fermenting strain of *Saccharomyces cerevisiae* by deleting an ion homeostasis regulatory gene. Our results illustrate a general approach for rapidly tailoring existing strains to tolerate specific chemical stressors encountered during industrial bioconversion.

## Methods

### Compounds, initial screening, and IC_50_ determination

Compounds tested were purchased from Sigma (St Louis, MO). Cells of *S. cerevisiae* (MATα *pdr1*Δ::*natMX pdr3*Δ::*KI.URA3snq2*Δ::*KI.LEU2can1*Δ::*STE2pr*-*Sp_his5lyp1*Δ *his3*Δ1 *leu2*Δ0 *ura3*Δ0 *met15*Δ0), referred to as control strain, were grown in 96-well microtiter plates with 200 µL cultures at 30 °C in YPD (10 µg/mL in yeast extract (10 g/L) peptone (20 g/L) medium with 1 % glucose), with a drug or dimethyl sulfoxide (DMSO) control. Cell densities of individual cultures were measured by optical density at 600 nm (OD_600_) using a TECAN M1000 over a 48 h growth period. The specific growth rate was calculated using GCAT analysis software (https://gcat3-pub.glbrc.org/) [13]. When presented, IC_50_ values for growth inhibition were calculated from triplicate eight point dose curves and SigmaPlot 12.0. When presented, error bars are Mean ± Standard error of at least three replicates.

### Chemical genomic analysis

Chemical genomic analysis of [EMIM]Cl was performed as described previously [17, 18]. The tested yeast deletion collection had 4194 strains using the genetic background described in Andrusiak (2012) [19]. We screened [EMIM]Cl at a concentration of YPD. 200 µL cultures of the pooled, deletion collection of *S. cerevisiae* deletion mutants were grown with [EMIM]Cl or a DMSO control in triplicate for 48 h at 30 °C. Genomic DNA was extracted using the Epicentre MasterPure™ Yeast DNA purification kit. Mutant-specific molecular barcodes were amplified with specially designed multiplex primers [20]. The barcodes were sequenced using an Illumina HiSeq 2500 in Rapid Run mode. Three replicates of each condition ([EMIM]Cl vs DMSO) were sequenced. The barcode counts for each yeast deletion mutant in the presence of [EMIM]Cl were normalized against the DMSO control conditions to define sensitivity or resistance of individual strains. To determine a p value for each top sensitive and resistant mutant, we used the EdgeR package [21, 22]. Data was visualized in Spotfire 5.5.0 (TIBCO, USA). A Bonferroni-corrected hypergeometric distribution test was used to search for significant enrichment of GO terms among the top 20 sensitive deletion mutants [23].

### Proteomic analysis of [EMIM]Cl treated cells

For yeast proteomics, triplicate 10 mL of YPD + 0.25 % [EMIM]Cl or YPD were inoculated with the control strain to a starting OD_600_ of 0.01 and incubated at 30 °C with shaking at 200 rpm. 2 mL of each culture was harvested when they reached an OD_600_ of ~0.5 (mid log phase growth). Cells were pelleted at 10,000 rpm, the media removed, and stored at −80 °C until processing for proteome analysis.

Yeast cell pellets were resuspended in 6 M GnHCl (Sigma, St. Louis, MO) with 50 mM tris pH 8.0 (Sigma, St. Louis, MO), boiled for 5 min, and precipitated by adding methanol (Thermo Fisher Scientific, Pittsburgh, PA) to a final concentration of 90 %. The precipitate was centrifuged at 10,000 rcf for 5 min, decanted, and air dried. The protein pellet was resuspended in 8 M urea (Sigma, St. Louis, MO) with 100 mM Tris pH 8.0, 10 mM tris (2-carboxyethyl) phosphine (Sigma, St. Louis, MO), and 40 mM chloroacetamide (Sigma, St. Louis, MO). The resuspended sample was diluted to 1.5 M urea with 50 mM Tris pH 8.0. Trypsin was added to a final ratio of 1:20 (enzyme to protein) and the samples were incubated at ambient temperature overnight. Peptides were desalted over Strata-X cartridges (Phenomenex, Torrance, CA). Desalted peptides were dried in a speed vac and resuspended in 0.2 % formic acid (Thermo Fisher Scientific, Rockford, IL). Peptides were quantified with the Pierce quantitative colorimetric peptide assay kit (Thermo Fisher Scientific, Rockford, IL).

For each analysis, 2 µg of peptides were separated across a 30 cm, 75 µm i.d. column packed with 1.7 µm BEH C18 particles (Waters, Milford, MA). Mobile phase A was 0.2 % formic acid and B was 0.2 % formic acid, 70 % ACN, and 5 % DMSO (Thermo Fisher Scientific, Pittsburgh, PA). The gradient was 5–50 % B over 100 min followed by a 100 % B wash and re-equilibration with 0 % B. Eluted peptides were analyzed on a Thermo Fusion Orbitrap (Thermo Fisher Scientific, San Jose, CA). Orbitrap survey scans were performed at 60,000 resolution, followed by ion-trap ms/ms analyses of the most intense precursors (with z = 2–6) for less than 3 s and using a dynamic exclusion of 15 s. The maximum injection time for each ms/ms was 25 ms and the ion-trap resolution was set to turbo.

Peptides were identified and quantified from the MS data using the MaxQuant software suite with the Andromeda and MaxLFQ search and quantitation algorithms, respectively. Spectra were searched against a Uniprot human proteome and common contaminant database concatenated with the reverse sequences. Match between runs was toggled on with the default settings. Peptide and protein identifications were filtered to 1 % FDR, and proteins were quantified by the MaxLFQ algorithm using the default settings. Data was visualized in Spotfire 5.5.0 (TIBCO, USA). A Bonferroni-corrected hypergeometric distribution test was used to search for significant enrichment of GO terms among the top 15 sensitive/resistant deletion mutants with a p value of p < 0.01 [23].

### Agar diffusion assay

YPD and YP-Glycerol (2 %) agar plates were inoculated with one OD of control strain cells grown overnight. These were then allowed to dry before removing four 0.5 cm diameter plugs from the plates. 50 µL of a 100 mg/mL solution of [EMIM]Cl in ddH2O was then placed into each hole and allowed to diffuse until the hole was dry. The plates were then grown for 24 h at 30 °C. A 1 % agar, 2 % carbon source, 1 % Triphenyl tetrazolium chloride (TTC) overlay was then placed over the cells and allowed to grow for 24 h for visualization.

### Microscopy of yeast mitochondria

Mid-log cultures of the control strain were used to inoculate a dose cure of [EMIM]Cl (0–1 %), and grown over night at 30 °C. After 18 h, the cultures were washed with 1X PBS and stained with SYTO18 (10 mM in HEPES; Life Technologies, USA). Cells were visualized fluorescence and GFP filter set. Photos were processed in Adobe Photoshop CC (Adobe, USA).

### Determination of mitochondrial membrane potential

To determine changes in mitochondrial membrane potential induced by ILs, we used FACS analysis of DiOC_6_(3) treated cells. 2 µL of log phase cells of the control yeast were added to 200 µL YPD with 0.25 % [EMIM]Cl, 200 µg/mL valinomycin, 170 µM antimycin a, 10 µg/ml benomyl, or solvent controls (water/DMSO) in triplicate. The cells were incubated for 4 h at 30 °C, pelleted at 3000 rpm, and the supernatant removed. Pellets were suspended in 10 mM HEPES buffer + 5 % glucose (pH 7.4) with 200 nM of DiOC_6_(3) (Life Technologies, Carlsbad, CA, USA), and incubated at RT for 30 min. Cells were diluted with HEPES buffer to appropriate density for FACS analysis. Green fluorescence of cells was quantified using a Guava EasyCyte (EMD Millipore, Billerica, MA, USA), and analysis GuavaExpress Pro software.

### Deletion of genes

To delete *PTK2* in Y133, we amplified the *PTK2* deletion cassette from the yeast knock out collection using flanking primers designed from published deletion primer sequences [24]. Following PCR clean up, we transformed the PCR product into Y133, and selected resistant colonies on YPD + G418 agar. We confirmed the deletion of *PTK2* by PCR using the confirmation primers described in [24].

### Overexpression of *PMA1*

We used the MoBY-ORF 2.0 version of *PMA1* expressed via a 2µ plasmids under its native promoter [25]. Y133 was transformed with either pPMA1 or a blank vector via high-efficiency transformation protocol [26], and successful transformants were identified on YPD + G418 agar medium. IIL sensitivity tests of the overexpression mutant were performed using an 8-point dose curve in YPD + G418 medium on a TECAN M1000 plate reader (TECAN, USA).

### Determining pH effects

To determine the effects of pH on [EMIM]Cl toxicity, triplicate wells of 198 µL of YPD medium at pH 6.5 or pH 5.0 (adjusted with 1 N HCl) ± 1 % [EMIM]Cl was incoculated with 2 µL of log phase cells of either Y133 or Y133-IIL and were grown for 48 h at 30 °C in a TECAN M1000 with growth measured every 15 min.

### Growth and sugar conversion experiments

To test the effects of [EMIM]Cl on fermentation, three 25 mL flasks were prepared with a YPXD (2 % glucose/2 % xylose) + 1 % [EMIM]Cl, and three with only YPXD. Flasks were inoculated with rinsed Y133 or Y133 *ptk2*∆ cells to bring the initial OD_600_ to approximately 0.1. The flasks were grown aerobically for 72 h with agitation at 30 °C. 1 mL samples were taken every 24 h. Initial and daily samples were measured for OD_600_ and submitted for HPLC analysis to quantify sugar consumption and ethanol production. To test the effects of [BMIM]Cl, twelve 25 mL anaerobic flasks and 12 10 mL aerobic tubes were prepared with a YPXD (2 % glucose/2 % xylose) + 1 % [BMIM]Cl. Six tubes and six flasks were brought to a pH of 6.5 and the remainder were brought to a pH of five with HCl. For each condition, (aerobic, pH 5 and 6.5, anaerobic pH 5 and 6.5) three flasks or tubes were inoculated with rinsed Y133 or Y133 *ptk2*∆ cells to bring the initial OD_600_ to approximately 0.1. One replicate of the Y133, anaerobic, pH 6.5 was lost for the 72 h sample point because of contamination. The tubes were grown for 72 h with agitation aerobically at 30 °C while the flasks were grown anaerobically for 72 h with agitation at 30 °C. 1 mL samples were taken every 24 h. Initial and daily samples were measured for OD and submitted for HPLC analysis to quantify sugar consumption and ethanol production.

## Results

### Chemical genomics predicts [EMIM]Cl affects mitochondria

**Fig. 2:**
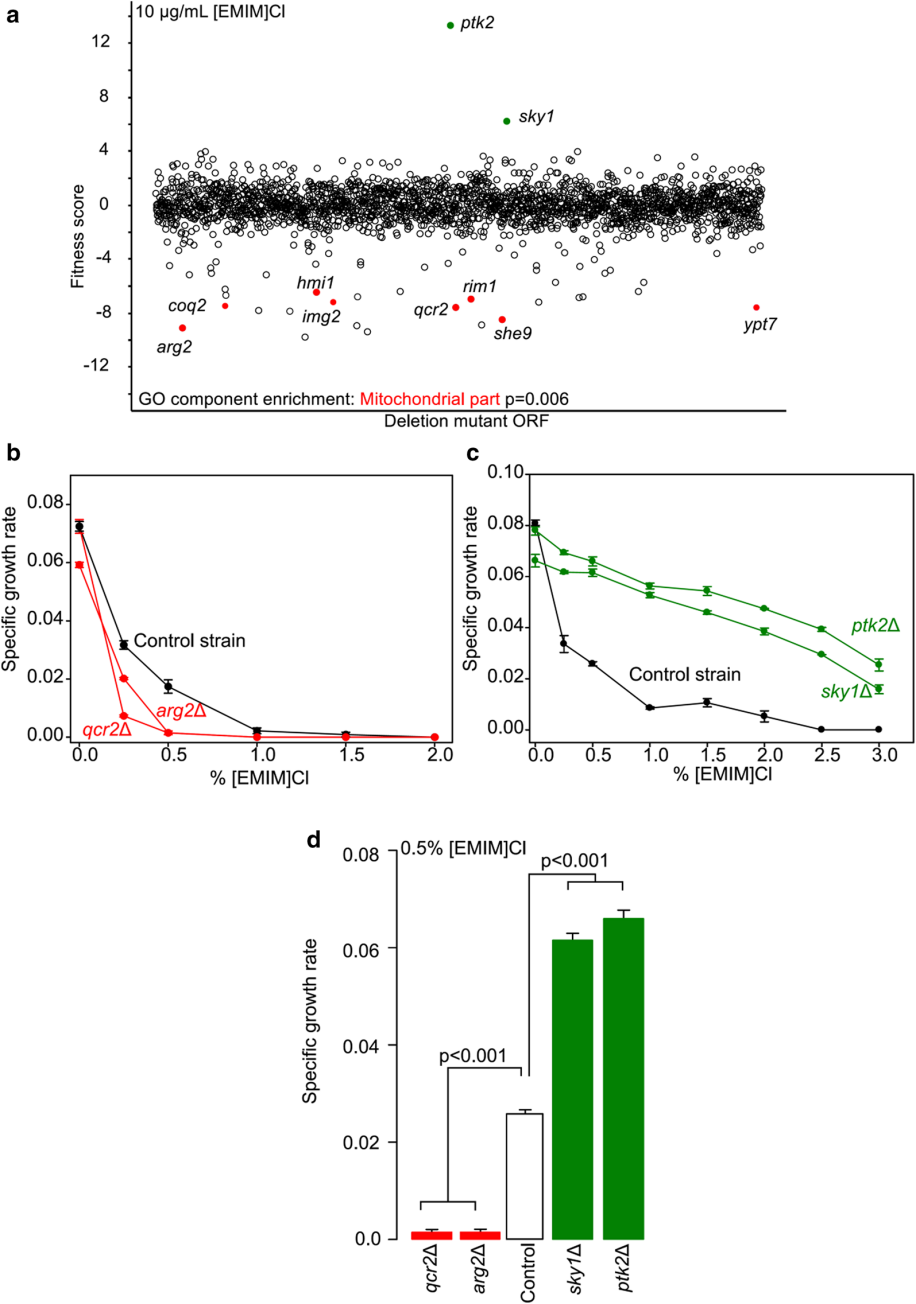
Chemical genomic profiling of [EMIM]Cl reveals mitochondrial genes are highly sensitive. Of the top 20 most significantly sensitive deletion mutants grown aerobically in YPD with 10 µg/mL [EMIM]Cl, eight were annotated to the mitochondrion (**a**). We tested the individual sensitivities of the top two most significantly sensitive and resistant mutants compared to the control strain (**b, c**) using an eight point dose curve. Mutants of *ARG2* and *QCR2* had significantly lower growth in 0.5 % [EMIM] Cl compared to the WT, whereas mutants of *PTK2* and *SKY1* grew significantly better (**d**). (n = 3, Mean ± S.E)

To identify targets of the IIL [EMIM]Cl, we undertook chemical genomic profiling using a panel of >4000 yeast non-essential gene deletion strains (Fig. 2a). Our screen identified 220 gene deletion mutants that were significantly responsive to [EMIM]Cl (10 µg/mL) (Additional file 1). Among the top 20 sensitive mutants, we found gene ontology (GO) enrichment (p < 0.01) for genes that encode mitochondrial proteins *(e.g*. *ARG2*, *COQ2*, *HMI1*, *IMG2*, *QCR2*, *RIM1*, *SHE9*, *YPT7*); thus, [EMIM]Cl may affect mitochondrial function (Fig. 2a). We individually examined the growth of the two top-ranked sensitive mutants (*QCR2*, *ARG2*) and confirmed that each displayed significantly greater (p < 0.01) sensitivity to [EMIM]Cl relative to the control strain (Fig. 2b, d).

Resistant mutants uncovered by chemical genomics can identify targets for rational engineering of resistance. The top resistant deletion mutant was *PTK2*, a putative serine/threonine protein kinase involved in regulation of ion transport across the plasma membrane, particularly polyamine cations [27, 28]. This mutant had a 12-fold increased (p = 1e^−74^) fitness in the presence of [EMIM]Cl, indicating greater growth than all other strains. The second most significant resistant strain was a deletion mutant of *SKY1* (fold change = 4.5, p = 1e^−21^), which is functionally similar to *PTK2* and is a protein kinase that also regulates proteins involved in cation homeostasis and polyamine cation uptake [27, 29]. We confirmed the resistance of these gene deletions individually, and the *PTK2* and *SKY1* deletions exhibited significantly (p < 0.01) higher [EMIM]Cl tolerance than the control strain (Fig. 2c, d). Finally, we correlated the chemical genomic profile of [EMIM]Cl to existing chemical genomic datasets [17] and found that it highly correlated with valinomycin (p < 0.001), a neutral ionophore that collapses K^+^ gradients across the mitochondrial membrane [30]; however, previous genome-wide studies have not shown that deletion of *PTK2* or *SKY1* confer significant resistance to valinomycin [17], which suggests [EMIM]Cl has a different mechanism of action.

### Chemical proteomics confirms toxic effects of IILs on mitochondria

**Fig. 3:**
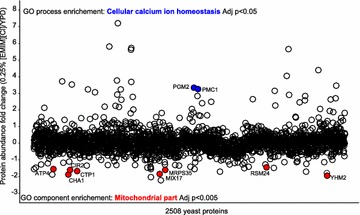
[EMIM]Cl treatment affects mitochondrial protein levels. Protein abundance and identity of yeast grown in the presence of [EMIM]Cl normalized against a solvent control demonstrates of the top 20 most depleted proteins, eight were annotated to the mitochondrial part. Among the most significantly (p < 0.01) more abundant proteins in the presence of [EMIM]Cl, two were specifically involved in calcium ion homeostasis (*blue*). (n = 3)

These chemical genomics data, as well as pervious reports [12], suggest that [EMIM]Cl may be toxic to mitochondria. As a validation, we next tested the yeast proteome response of the strain to [EMIM]Cl treatment. We grew cells to mid-log with or without 0.25 % [EMIM]Cl and then measured the levels of cellular proteins using high-throughput quantitative proteomics [31]. We found that among 729 proteins that changed levels significantly (p < 0.01) in response to the IIL (Additional file 2), many mitochondrial proteins were significantly decreased in concentration compared to untreated cells (p < 0.005; Fig. 3). Among these were two proteins involved in mitochondrial citrate transport (Ctp1p, Ymh2p) and two mitochondrial ribosome proteins (Mrps35p, Rsm24). Among top proteins with increased abundance, we observed enrichment for proteins involved in small molecule catabolism (p < 0.001) driven by Thi20p, Pgm2p, Car2p, Xks1p, Uga2p and Gad1p. More specifically, we observed enrichment (p < 0.05) for two proteins involved in calcium ion homeostasis (Pgm2p and Pmc1p). Taken together with the sensitive deletion mutants discovered in the chemical genomic profile, these data suggest IILs are toxic to the mitochondria, and specifically ion transport across the mitochondrial membrane.

### [EMIM]Cl disrupts mitochondrial membrane potential

**Fig. 4:**
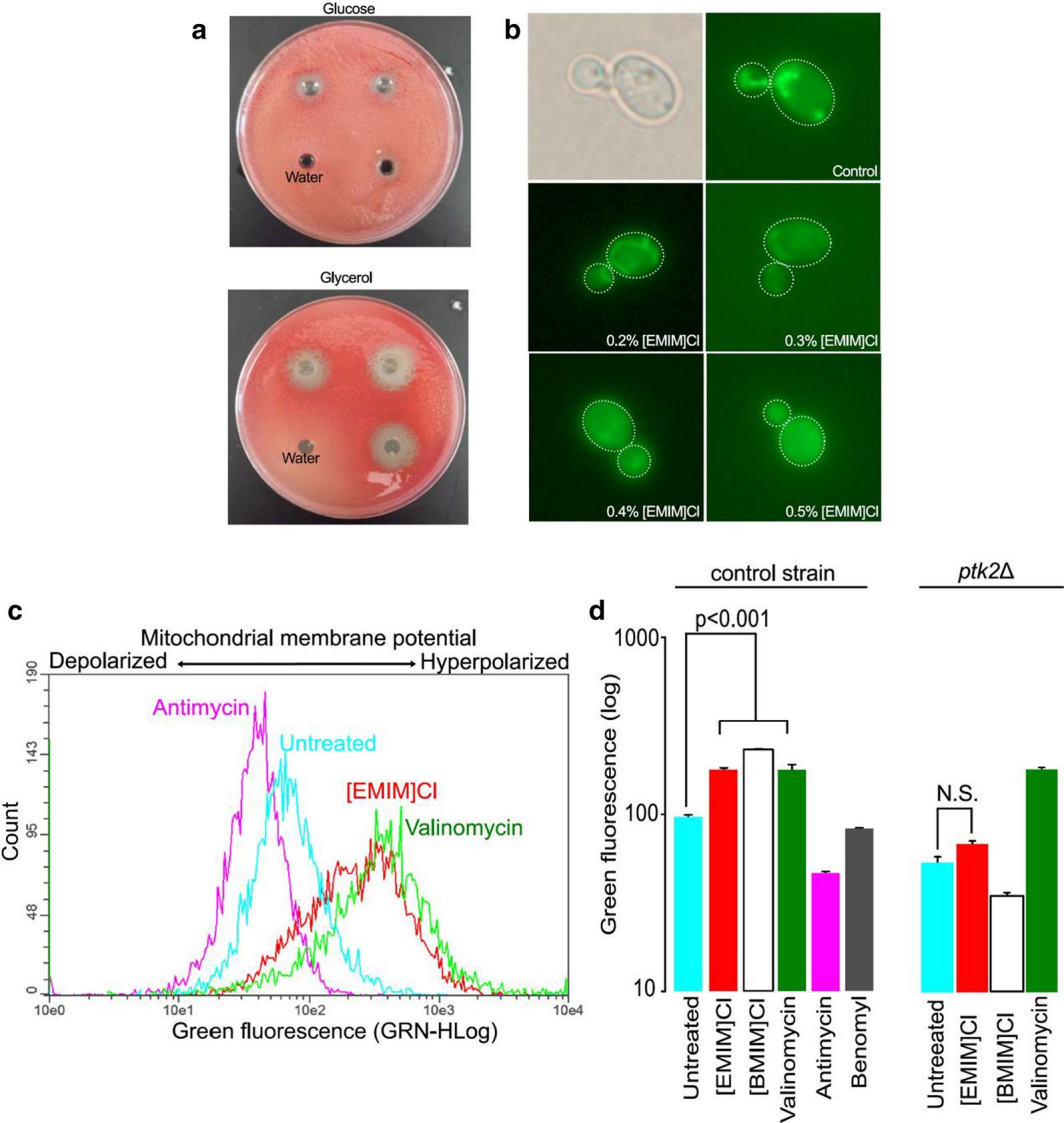
Effects of [EMIM]Cl on respiration, mitochondrial structure, and membrane potential. Zones of inhibition caused by [EMIM]Cl on yeast grown on either glycerol or glucose (**a**). Dose dependent disappearance of yeast mitochondrial structure (tubular structures stained with SYTO18) in the presence of [EMIM]Cl (**b**). [EMIM]Cl treatment at sub lethal doses (0.25 %) causes increases DiOC_6_(3) fluorescence, as does the ionophore valinomycin (**c, d**). The uncoupling agent antimycin is included as a positive control, and the tubulin poison benomyl is included as an inhibitor with a mode of action unrelated to the mitochondrion. DiOC_6_(3) fluorescence of the *PTK2* mutant when treated with [EMIM]Cl, [BMIM]Cl, valinomycin or control (**d**)

If IILs were toxic to mitochondria, their effects would be expected to be enhanced during aerobic growth on non-fermentable substrates, which requires mitochondrial-dependent respiration. We found that IILs were indeed more toxic during cell growth on glycerol, which requires respiration, compared to growth on glucose, which does not (Fig. 4a).

Using microscopy, we next explored the effect of IILs on mitochondria. Cultures treated with [EMIM]Cl displayed a dose-dependent effect on mitochondrial structure, as determined with the stain SYTO18, which preferentially binds yeast mitochondrial nucleic acids (Fig. 4b). Untreated cells exhibited normal, tubular mitochondrial morphology; whereas at high doses, discrete morphology disappears, and the SYTO18 signal appeared diffused through the cell, suggesting a breakdown of mitochondrial integrity and release of mitochondrial nucleic acids. Finally, we used FACS analysis with the fluorescence stain DiOC_6_(3), which accumulates in the mitochondrial membrane as a function of membrane potential. With a 4-h treatment, the known ionophore valinomycin caused hyperpolarization of the mitochondrial membrane, whereas the membrane potential uncoupler antimycin resulted in depolarization relative to the DMSO-only control (Fig. 4c, d). With [EMIM]Cl treatment, we observed a significantly (p < 0.01) increased fluorescence shift of DiOC_6_(3), indicating a hyperpolarized mitochondrial membrane potential and thus greater dye uptake, similar to valinomycin (Fig. 4c, d). The tubulin poison benomyl was included as control agent that causes cell death through a mechanism unrelated to the mitochondria, as expected this compound did not alter membrane potential. Mitochondrial hyperpolarization can lead to ROS production and ultimately apoptosis in yeast [32]. Although mitochondria are not required for sugar fermentation to ethanol, they are required for fatty acid biosynthesis and other essential growth processes during anaerobiosis [33]. We found that the effect of [EMIM]Cl, but not valinomycin, on mitochondrial membrane hyperpolarization was alleviated in the *PTK2* deletion mutant (Fig. 4d). This result suggests that influx of [EMIM]Cl is different than that of valinomycin, and possibly that the *PTK2* deletion mutant decreases uptake of toxic [EMIM]^+^ cation, similar to how uptake of other cations (e.g. spermine, tetramethylammonium) is reduced by the *PTK2* deletion.

### Engineering IIL tolerance through an understanding of toxicity

Our findings suggest that IIL affects mitochondrial function, and that deletion mutations known to decrease cation influx can increase IIL tolerance. We identified two kinase regulators whose deletion decreases IIL toxicity, *PTK2* and *SKY1*. The products of both genes are known to enhance spermine uptake. As both spermine and IILs are weak bases, the role of *PTK2* and *SKY1* in IIL uptake may be similar to that in polyamine cation uptake. Spermine is transported by *TPO1, 2, 3,* and *4* in yeast, however none of these gene mutants were significantly responsive to [EMIM]Cl (Additional files 1, 2), which suggest IILs may have a different transporter regulated by *PTK2*.

As *ptk2∆* was the most resistant mutant in both the initial screen and the validation tests, we focused on this gene for engineering. *PTK2* and *SKY1* have a well-documented negative genetic interaction (deletion of both genes has a synergistic, negative effect on cell fitness) [34, 35], which would potentially throttle conversion rates in IIL produced hydrolysates. Further, the IIL tolerance of the *PTK2* mutant alone was substantially higher (>2 % [EMIM]Cl) than levels of residual IILs that are found in IIL produced hydrolysates [8]. For these reasons, we chose not to delete both genes.

**Fig. 5:**
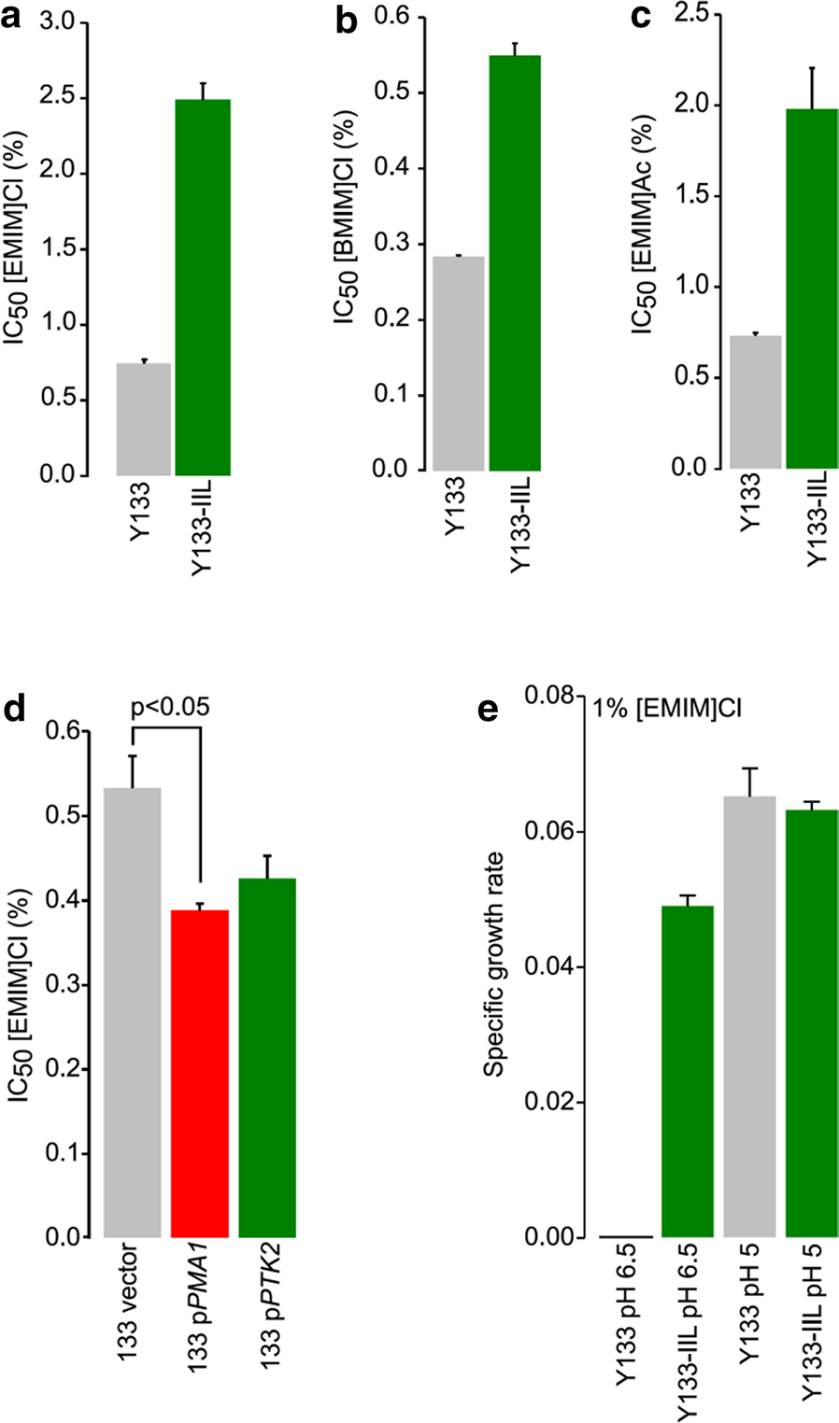
The effect of IILs on cell growth in the background strain (Y133) or *PTK2* mutant (Y133-IIL). IC_50_ values were determined for each xylose fermenting yeast strain grown in YPD containing various concentrations of [EMIM]Cl (**a**), [BMIM]Cl (**b**), or [EMIM]Ac (**c**). In (**d**), Y133 was transformed with the indicated plasmids, and effects on IC_50_ for [EMIM]Cl using the resulting transformants were assessed. To examine pH dependence on IIL toxicity, specific growth rates of the Y133 and Y133-IIL strains cultured in YPD media containing 1 % [EMIM]Cl at pH 5 or 6.5 (**e**). Mean ± S.E

For conversion of lignocellulose to fuels and useful chemicals by *S. cerevisiae*, xylose-conversion remains a key challenge. Strains have been engineered and evolved for robust xylose fermentation [14, 36, 37], but their use to convert IIL-derived hydrolysates has not been reported. To test whether IIL-tolerance traits identified using lab strains can be used to engineer industrially relevant strains, we deleted *PTK2* in a isolate of *S. cerevisiae* engineered for xylose-fermentation (Y133) [14]. The half-maximal growth inhibition (IC_50_) of [EMIM]Cl in Y133 yeast was 0.76 %, whereas the Y133 *ptk2Δ* mutant (hereforth called Y133-IIL) had an IC_50_ of 2.4 % [EMIM]Cl (Fig. 5a). This improved tolerance was less dramatic than we observed in the control strain, but was still well above the amounts of IIL that would be expected in IIL hydrolysates. Importantly, this modification similarly conferred tolerance to other IILs, [BMIM]Cl and [EMIM]Ac (Fig. 5b, c), suggesting that toxicity of most or all IILs can be reduced by deletion of *PTK2*.

*PTK2* is known to activate the essential proton efflux pump Pma1p by phosphorylation [38]. We found increased expression of *PMA1* caused a significant decrease in [EMIM]Cl tolerance (Fig. 5d, p < 0.01). Overexpression of *PTK2* also reduced [EMIM]Cl tolerance, but not significantly. This suggests that proton efflux by Pma1p may be coupled with influx of the toxic IIL cation, and that decreasing the activity of Pma1p by deletion of *PTK2* can confer resistance to IILs. Interestingly, we observed a strong pH effect on IIL toxicity. At near neutral pH (pH 6.5), growth inhibition by [EMIM]Cl was greater towards Y133, whereas there was not a significant difference in growth between Y133 and the Y133-IIL at a lower pH (pH 5.0) (Fig. 5e), perhaps because the lower pH decreases proton efflux by mass action. Uptake of polyamine cations like spermine are highly pH dependent; their uptake increases at higher pH [39] and IILs may be subject to a similar effect.

### Fermentative performance of IIL tolerant yeast

**Fig. 6:**
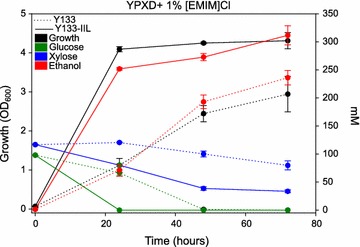
Growth (*black*), sugar consumption (glucose, *green*; xylose, *blue*), and ethanol production (*red*) of Y133-IIL (*solid lines*) vs Y133 (*dashed lines*) in YPXD media with 1 % [EMIM]Cl at under aerobic conditions at pH 6.5. (n = 3, Mean ± S.E, * p < 0.05)

**Fig. 7:**
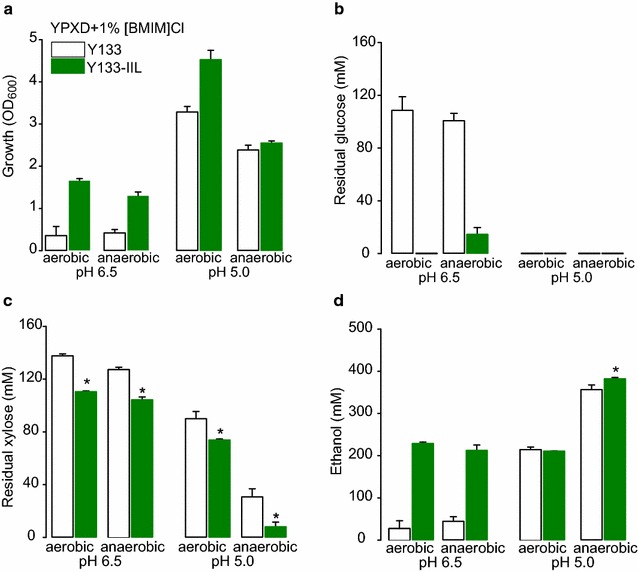
Final growth and metabolites analysis after of Y133 and Y133-IIL in the presence of [BMIM]Cl. Growth (**a**), glucose and xylose consumption (**b, c**) and ethanol production (**d**) after 72 h of culture under aerobic and anaerobic conditions at pH 6.5 or pH 5.0. (n = 3, except n = 2 for Y133 pH 6.5, Mean ± S.E, * p < 0.05)

The fermentative capacity of the engineered, IIL-tolerant yeast versus the parent strain is the best test of the chemical genomics-guided biodesign. Y133-IIL had greater growth and sugar conversion in the presence of 1 % [EMIM]Cl than the background strain under aerobic conditions at pH 6.5 (Fig. 6). We further tested the effects of both pH and oxygen on the performance of Y133 and Y133-IIL (Fig. 7a–d, Additional file 3). Because Y133-IIL exhibited cross resistance to all three IILs tested, we chose to assess fermentation performance in the presence of [BMIM]Cl, the most toxic IIL and one of particular interest for its lignocellulose deconstruction properties [11]. The greatest IIL toxicity occurs near neutral pH under aerobic conditions in the WT strain, where the Y133-IIL strain converted significantly more glucose and xylose to ethanol (Fig. 7b–d). At pH 5.0, the differences between the two strains were less dramatic, but the Y133-IIL strain still converted significantly more xylose to ethanol (Fig. 7c, d, p < 0.05), even under anaerobic conditions. Of note, in the absence of [BMIM]Cl, Y133-IIL performed equivalently to Y133 (if not slightly worse in some conditions) in terms of sugar conversion (Additional file 4); thus, the *PTK2* deletion alone has no generally positive effect on growth, but confers a specific advantage in the presence of IILs. Although IIL toxicity can be alleviated at reduced pH and in anaerobic conditions, the *PTK2* modification still confers an advantage that results in greater ethanol production.

### A proposed mechanism of IIL toxicity and tolerance

**Fig. 8:**
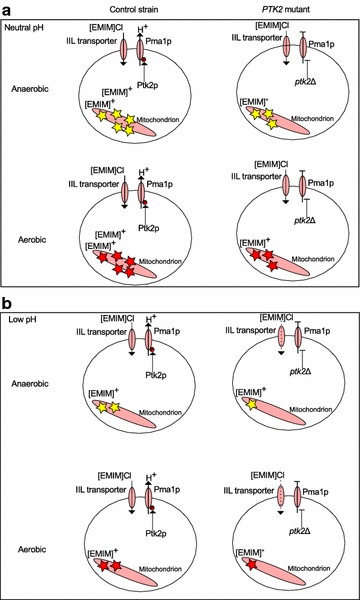
A model for IIL toxicity and resistance. We propose the model of imidazolium IIL toxicity. In the presence of IILs at near neutral pH (**a**), cells pump out protons via Pma1p, which is coupled with import of the [EMIM]^+^ cation that results in hyperpolerization of the mitochondrial membrane. *PTK2* activates Pma1p via phosphorlaytion. Deletion of *PTK2* alleviates this by reducing Pma1p activity, and thus [EMIM]Cl influx. The effects of mitochondrial perturbation are more acute in aerobic conditions (*red stars* vs *yellow stars*), where mitochondria are more active. At lower pH (**b**), [EMIM]Cl import is lessened, similar to the polyamine cation spermine, which is alone regulated by *PTK2*

Based on these results, we propose the following model for IIL toxicity in yeast and its modulation by oxygen level and extracellular pH (Fig. 8). IILs induce increased proton efflux via Pma1p (activated by *PTK2*), which is coupled with import of the toxic imidazolium cation, similar to the role of *PTK2* in uptake of the polycation spermine [28, 29]. Once inside the cell, the IIL cation interacts with mitochondria leading to hyperpolarization of the mitochondrial membrane [32]. Decreasing proton efflux by deletion of *PTK2* lessens accumulation of the IIL cation in cells and hence its interaction with mitochondria. The exact target of the imidazolium cation remains unclear. It may bind a specific mitochondrial enzyme or insert into the mitochondrial membrane to affect function of membrane-associated enzymes, leading to altered membrane potential. Regardless of the target, we predict this toxic effect will be more acute during aerobic respiration (Fig. 8, top half of each panel), which requires mitochondrial activity. ILL toxicity is increased at a higher pH (Fig. 8 top panel) because cation uptake is greater, similar to what has been observed for spermine transport [39]. When *PTK2* is deleted, activation of Pma1p is reduced, leading to less proton efflux and less transport of the toxic imidazolium cation into the cell (Fig. 8, right).

## Discussion

Using chemical genomic and proteomic profiling, we identified a potential mechanism for toxicity of imidazolium ILs in yeast. These agents damage mitochondrial function, apparently by inducing hyperpolarization of the mitochondrial membrane. In yeast, hyperpolarization of the mitochondrial membrane can ultimately lead to ROS production and apoptosis [32]. Future studies using GFP-fused mitochondrial proteins will be useful in determining the effect of IILs on specific proteins and gaining better resolution of mitochondrial morphological changes that occur upon exposure to IILs. IIL accumulation appears to differ from that of the K^+^ ionophore valinomycin, as IIL-induced hyperpolarization can be alleviated by deletion of the kinase regulator of ion homeostasis *PTK2*, whereas *ptk2∆* has no effect on valinomycin-mediated hyperpolarization (Fig. 4d). This result is consistent with Pma1p-coupled influx of the IIL cation; valinomycin is a neutral molecule and its intercellular accumulation would not be stimulated by proton efflux. Our model posits an IIL-specific transporter, but further work will be required to identify the transporter. IIL toxicity can be lessened at lower pH. Further, as toxicity appears to result from impaired mitochondrial function, growth under strict anaerobic conditions also lessens IIL toxicity. Nonetheless, mitochondrial function remains essential for cell viability even under strict anaerobic conditions, for instance for fatty acid biosynthesis [33]; thus, the *PTK2* modification increases IIL tolerance and sugar utilization even during anaerobic fermentation at low pH (Fig. 7b).

IIL-based deconstruction methods hold significant promise for a feedstock-agnostic hydrolysates to feed lignocellulosic biorefineries. These IIL-based methods appear to be equivalently robust for deconstruction of both grasses and wood [40]. IILs in particular show promise for their ability to generate relatively pure sugar and lignin streams [6]. Thus, understanding the mechanisms of IIL toxicity in fermentative microbes is key to generating microbial strains engineered for IIL-based hydrolysates. Because removing residual IILs inevitably will confer an added cost in hydrolysate production, rational engineering of IIL-tolerant fermentative microbes can decrease the overall cost of IIL-based lignocellulosic biofuels and products.

Our results illustrate a general paradigm by which chemical genomics can enable rapid strain design in response to emerging bioconversion technologies. Both lignocellulose deconstruction technologies and the resulting landscape of fermentation inhibitors continue to evolve. Continued strain development will be necessary to keep pace with these new technologies and chemical stressors like IILs. Further, different industrial settings often necessitate use of different strain backgrounds; thus, it will be important that advantageous traits can be introduced rationally into diverse strain backgrounds. Our chemical genomics approach enables identification of such readily exploited traits for rational engineering. As our discovery system is based on *S. cerevisiae*, the primary lignocellulosic biorefinery microbe, the gene identified can be directly modified in other yeast strains to rapidly tailor proven strains for new hydrolysates.

## Conclusions

Chemical genomics-guided biodesign for strain engineering can also be applied to other bioproducts in addition to ethanol. Drugs, green chemicals, and next-generation fuels can be produced by yeast and other engineered microbes, and many of these end-products can be toxic to the biocatalyst microbe. The chemical genomics approach is a general way to define their mechanism of toxicity and discover means to engineer tolerance and improve their production. This approach is not limited to yeast; genome-wide mutant and overexpression collections exist in a number of industrially relevant microbes, including *Escherichia coli* and *Zymomonas mobilis,* making the chemical genomics approach translatable to these microbes as well.

## Additional files

10.1186/s12934-016-0417-7 Significantly responsive [EMIM]Cl deletion mutants.
10.1186/s12934-016-0417-7 Significantly responsive protein abundance in [EMIM]Cl.
10.1186/s12934-016-0417-7 Fermentation profiles of Y133 and Y133-IIL in the presence of 1 % [BMIM]Cl at pH 6.5 and pH 5.0, and either aerobic or anaerobic conditions (n = 3, Mean ± S.E, except n = 2 for Y133 pH 6.5 anaerobic 72 h).
10.1186/s12934-016-0417-7 Final growth and metabolites analysis after of Y133 and Y133-IIL in the absence of [BMIM]Cl. Growth (**a**), glucose and xylose consumption (**b, c**), and ethanol production (**d**) after 72 h of culture in the presence of [BMIM]Cl at pH 6.5 and pH 5.0 in both aerobic and anaerobic conditions. (n = 3, Mean ± S.E).

## Abbreviations

IL: ionic liquid
IIL: imidazolium ionic liquid
YPD: yeast extract peptone dextrose
DMSO: dimethyl sulfoxide
