# Supplementary figures and images for: Mechanism of imidazolium ionic liquids toxicity in Saccharomyces cerevisiae and rational engineering of a tolerant, xylose-fermenting strain
Source: Microb Cell Fact. 2016 Jan 20;15:17. doi: 10.1186/s12934-016-0417-7 (PMC4721058; doi:10.1186/s12934-016-0417-7)

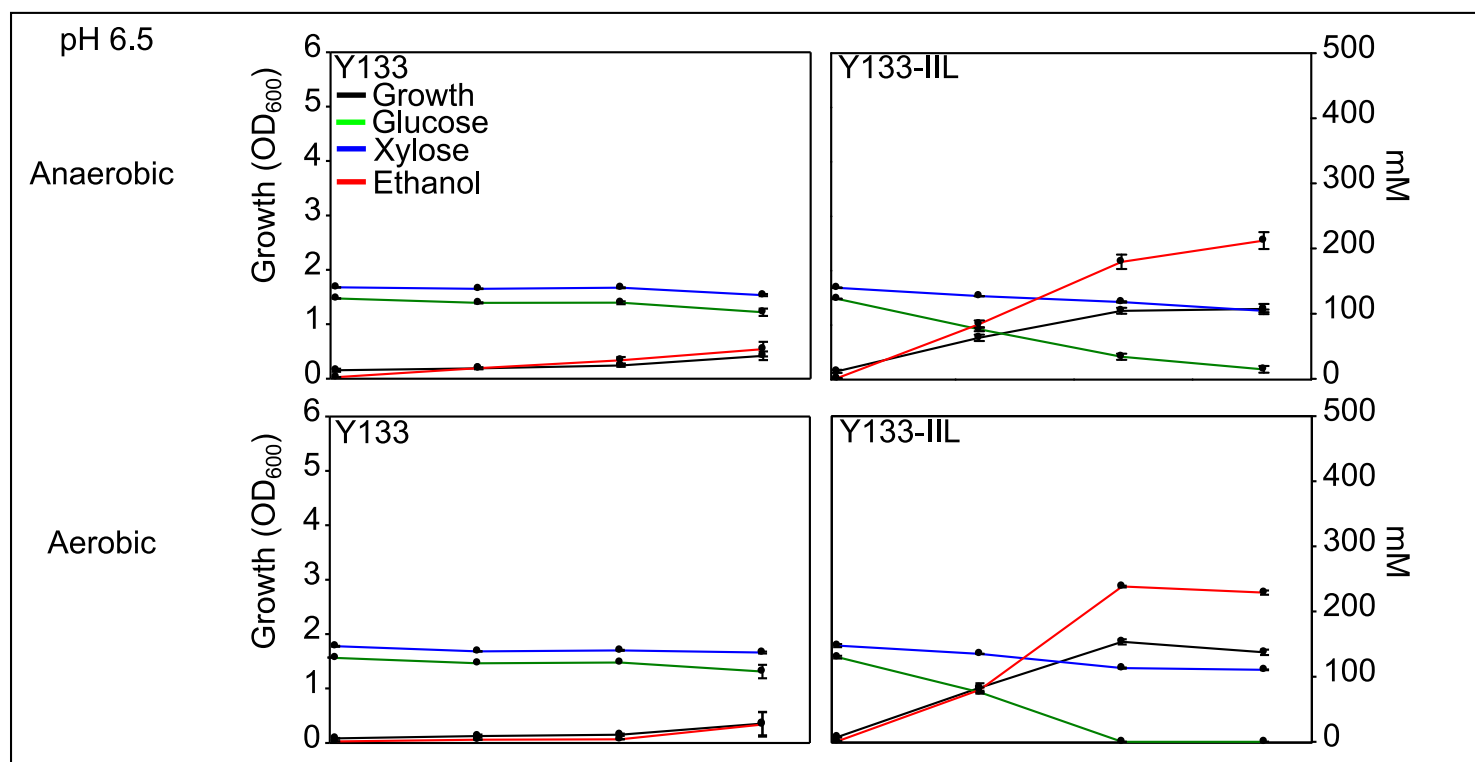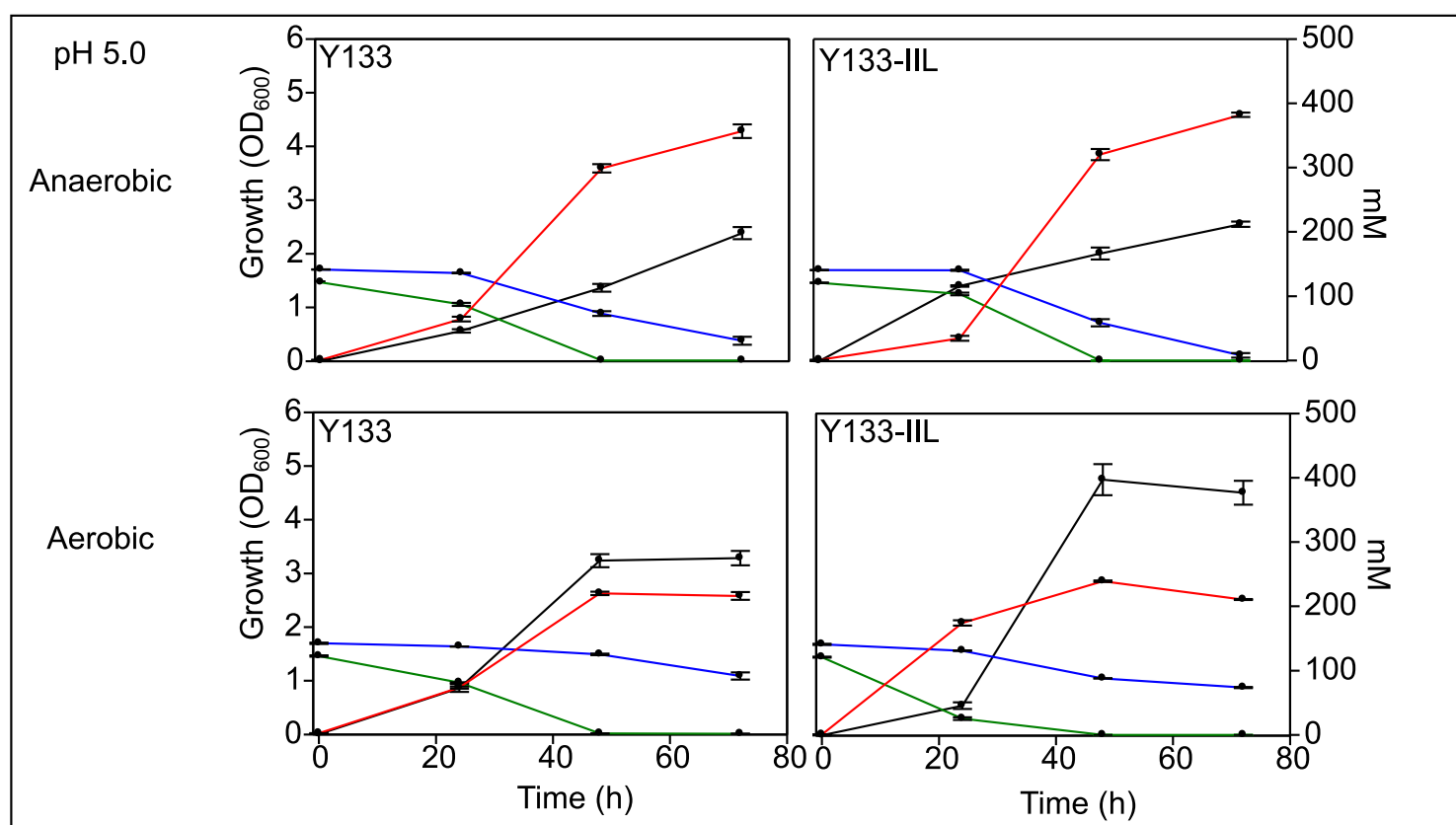

Supplement: Supplementary file 3 — 10.1186/s12934-016-0417-7 Fermentation profiles of Y133 and Y133-IIL in the presence of 1 % [BMIM]Cl at pH 6.5 and pH 5.0, and either aerobic or anaerobic conditions (n = 3, Mean ± S.E, except n = 2 for Y133 pH 6.5 anaerobic 72 h). [file 12934_2016_417_MOESM3_ESM.pdf]

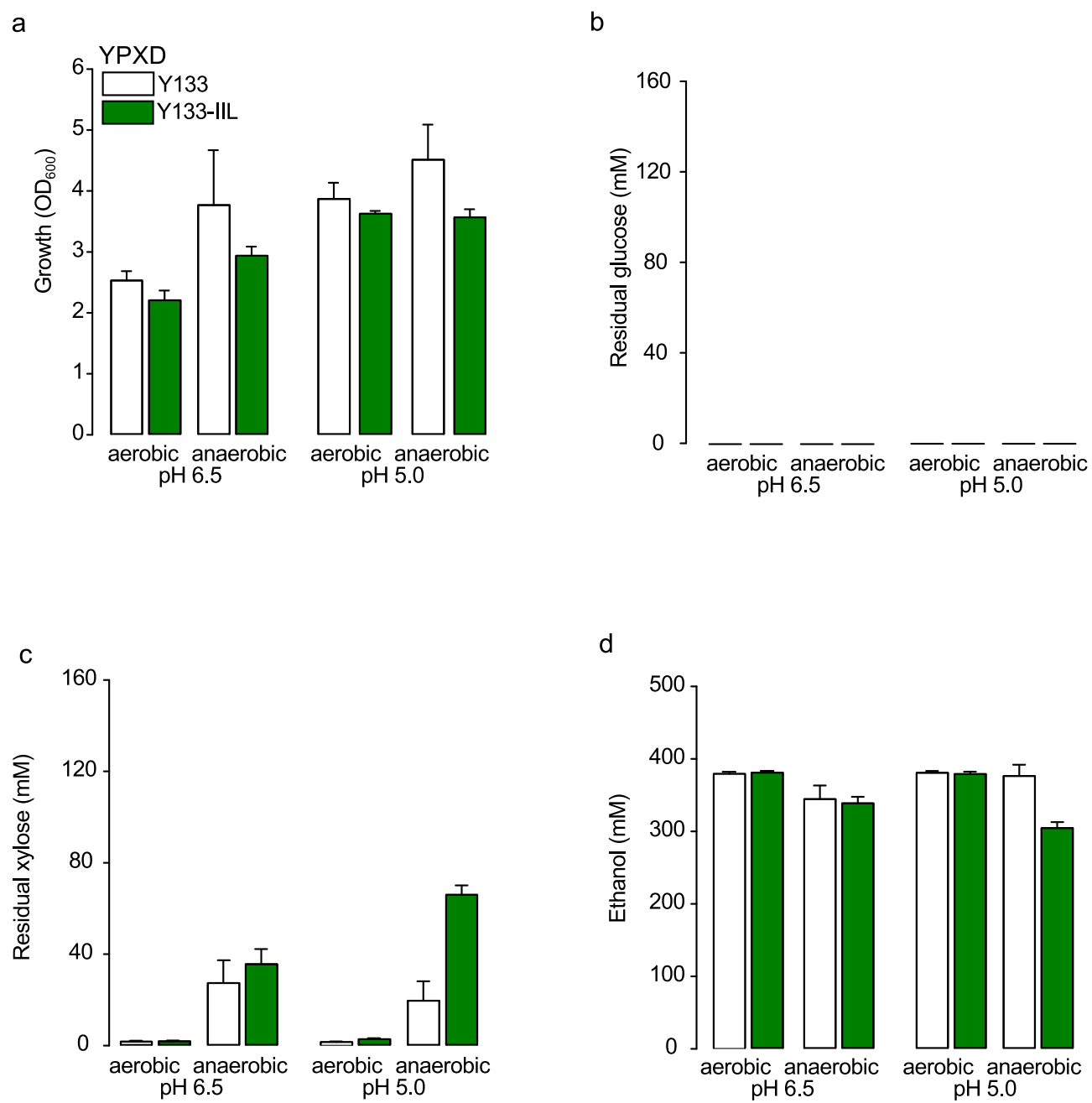

Supplement: Supplementary file 4 — 10.1186/s12934-016-0417-7 Final growth and metabolites analysis after of Y133 and Y133-IIL in the absence of [BMIM]Cl. Growth (a), glucose and xylose consumption (b, c), and ethanol production (d) after 72 h of culture in the presence of [BMIM]Cl at pH 6.5 and pH 5.0 in both aerobic and anaerobic conditions. (n = 3, Mean ± S.E). [file 12934_2016_417_MOESM4_ESM.pdf]
